# Association of Antipsychotic Polypharmacy and Two-Year All-Cause Mortality: A Population-Based Cohort Study of 33,221 Italian Continuous Users

**DOI:** 10.3390/jcm13072073

**Published:** 2024-04-03

**Authors:** Alberto Parabiaghi, Matteo Monzio Compagnoni, Barbara D’Avanzo, Giulia Caggiu, Alessia A. Galbussera, Mauro Tettamanti, Ida Fortino, Angelo Barbato

**Affiliations:** 1 Unit for Quality of Care and Rights Promotion in Mental Health, Istituto di Ricerche Farmacologiche “Mario Negri”-IRCCS, 20156 Milan, Italy; alberto.parabiaghi@marionegri.it (A.P.); angelo.barbato@marionegri.it (A.B.); 2 Unit of Biostatistics, Epidemiology and Public Health, Department of Statistics and Quantitative Methods, University of Milano-Bicocca, 20126 Milan, Italy; giulia.caggiu@unimib.it; 3 National Centre for Healthcare Research and Pharmacoepidemiology, University of Milano-Bicocca, 20126 Milan, Italy; 4 Laboratory for Assessing Quality of Care and Services, Istituto di Ricerche Farmacologiche “Mario Negri”-IRCCS, 20156 Milan, Italy; barbara.davanzo@marionegri.it; 5 Department of Mental Health and Addiction Services, ASST Lecco, 23900 Lecco, Italy; 6 Laboratory of Geriatric Epidemiology, Istituto di Ricerche Farmacologiche “Mario Negri”-IRCCS, 20156 Milan, Italy; alessia.galbussera@marionegri.it (A.A.G.); mauro.tettamanti@marionegri.it (M.T.); 7 Directorate General for Health, Lombardy Region, 00144 Milan, Italy

**Keywords:** pharmacoepidemiology, drug safety, antipsychotic monotherapy, antipsychotic polytherapy, all-cause mortality, metabolic side effects, drug utilization research

## Abstract

**Background:** Differences in survival between patients treated with antipsychotic monotherapy vs. polytherapy are debated. This study aimed to examine the association of antipsychotic polytherapy with 2-year all-cause mortality in a population-based cohort. **Methods:** Data were retrieved from healthcare databases of four local health units of Lombardy, Italy. Subjects aged 18–79 years who received continuous antipsychotic prescriptions in 2018 were identified. Overall survival among patients with antipsychotic monotherapy vs. polytherapy was compared. A multivariate Cox PH model was used to estimate the association between antipsychotic therapy, or antipsychotic use (continuous vs. non-continuous), and all-cause mortality. Adjustments were made for the presence of metabolic disturbances, total antipsychotic dosage amount (olanzapine equivalent doses), age, and sex. **Results:** A total of 49,875 subjects receiving at least one prescription of antipsychotics during 2018 were identified. Among the 33,221 patients receiving continuative antipsychotic prescriptions, 1958 (5.9%) experienced death from any cause at two years. Patients with continuous antipsychotic use had a 1.13-point increased mortality risk compared with non-continuous users. Patients treated with antipsychotic polytherapy showed an adjusted mortality risk increased by 17% (95% CI: 2%, 33%) compared to monotherapy. **Conclusions:** The study highlights the potential risks associated with antipsychotic polypharmacy, emphasizing the importance of optimizing drug prescriptions to improve patient safety and reduce mortality rates in individuals receiving antipsychotic therapy.

## 1. Introduction

Antipsychotic (AP) drug prescription is the gold standard therapy for schizophrenia and other psychotic disorders. Although they are usually indicated to treat psychosis, in recent years, their use has been expanded to include bipolar and depressive disorders, and off-label use has become common for treating behavioral symptoms of dementia, anxiety disorders, and obsessive–compulsive disorder, disturbing behavior in mental retardation and pervasive developmental disorders [1].

Antipsychotic monotherapy (APM), particularly with second-generation antipsychotics (SGAs), is the most suggested treatment modality as it allows one to evaluate the clinical response and helps the simplification of medication regimen facilitating adherence, reducing the risk of serious adverse effects and reactions [2,3,4,5,6,7]. However, despite most recommendations, combinations of antipsychotics have been widely used over the last few decades for treating insufficient or no clinical response or intolerance to initial monotherapy in routine clinical care [8,9,10]. This trend toward antipsychotic polytherapy (APP) has for long garnered concern from the research community, primarily due to the scarcity of evidence supporting its efficacy, and safety concerns highlighted by increased adverse reactions, drug–drug interactions, and the necessity for additional medications to counter emerging side effects [11,12,13,14]. Additionally, the economic implications of APP, incrementing healthcare costs associated with the treatment of psychotic disorders, further complicate its justification [13,14]. Thus, the persistent clinical use of APP over the years has highlighted a significant gap between evidence, guidelines, and real-world treatment practices [8,15,16,17,18]. This enduring discrepancy underlines the complex dynamics within psychiatric care where, despite clear recommendations and evidence supporting APM, the prevalence of APP has continued to rise, whereas efforts of deprescribing have been reported in few studies [19,20,21].

Leading up to the most recent years, there have been emerging suggestions that specific combinations of antipsychotics could offer benefits under certain conditions [22,23]. As early as 2009, a meta-analysis of randomized controlled trials by Correl et al. highlighted the intricate challenges in directly comparing APP with APM, suggesting possible advantages of APP [24]. Notable examples have later included the combination of aripiprazole with clozapine, which has been reported to effectively reduce treatment side effects and residual symptoms and lower the risk of psychiatric rehospitalization [25,26]. But this evolution in perspective did not stop there, as recent developments indicate a shifting paradigm, questioning the long-standing preference for APM and further exploring the potential benefits of APP within specific clinical scenarios. This evolving viewpoint is supported by several observational studies. Among these, a recent study found no significant difference in mortality risk between long-term APP and APM users among seriously mentally ill adults [27]. Additionally, epidemiological insights from a nationwide cohort of schizophrenic patients from Finland pointed toward potential benefits of APP, advocating for a more detailed investigation into its safety and effectiveness [28]. Their findings argue against outright discouragement of APP in clinical guidelines without solid evidence of safety concerns [28]. Moreover, a comprehensive review on global AP use highlighted the diverse application rates of APP and its association with varying clinical practices and outcomes [29]. Thus, the recognition of APP extensive use in clinical practice, coupled with emerging evidence questioning the traditional bias against APP, underscores the necessity for further exploration into this topic. Given these evolving perspectives on antipsychotic treatment strategies, it is important to keep scrutinizing their potential impact on the most severe risks associated with psychotic disorders, underscoring the need to carefully evaluate therapeutic options.

Increased mortality and shorter life expectancy are significant concerns for those with psychotic disorders. A notable study encompassing 70,586 patients from four Italian regions in 2015 highlighted the severe mortality risk in schizophrenia patients, revealing a twofold increase in mortality compared to the general population [30]. This finding is consistent with broader evidence indicating excess mortality among psychotic patients, as further evidenced by a recent meta-analysis that reported a gender-pooled standardized mortality ratio (SMR) of 3.08 (95% CI: 2.88–3.31), showing a persistently high risk over the years [31]. Moreover, schizophrenia’s serious impact on life expectancy was underscored by another meta-analysis that associated the presence of this disorder with an average loss of 14.5 years of life expectancy (95% CI: 11.2–17.8), reinforcing the urgent need for targeted interventions [32]. The number of years of life lost, the higher mortality rate, and the lack of improvement over time call for the urgent need of interventions and initiatives to reduce this gap. Efforts should be made to improve the safety of psychopharmacological treatments and, more generally, to reduce mortality.

Indeed, the optimization of drug prescription is certainly one of the options on the table, as side effects of AP medications (i.e., weight gain, dyslipidemia, and glycemic disturbances) are among the potential causes of the observed mortality excess [33]. Furthermore, the concept of neuropsychiatric stress could offer valuable insights into more complex influences on mortality differences. In recent years, stress and metabolic disorders were identified as pivotal factors in the worsening and pathogenesis of mental disorders, suggesting a complex interplay between environmental stressors, cytokine activity, and chronic metabolic stress on mental health [34,35,36]. Thus, neuropsychiatric stress, stemming from chronic mental health disorders, could worsen psychiatric conditions, encouraging more APP use. This suggests a vicious cycle where increased APP use and the resultant metabolic health decline further aggravate neuropsychiatric stress, contributing to the observed mortality risk increase.

Finally, although lifestyle and genetics may contribute as independent risk factors of cardiometabolic dysfunction in psychotic disorders, antipsychotic treatment represents an important contributor, particularly for certain drugs and vulnerable patients. APP was shown to cause more side effects than APM, which is the main reason why most treatment guidelines suggest caution against it and why deprescribing has been considered a goal of treatment quality improvement [19,20]. However, a clear link between APP and excess mortality is still missing [27].

### Aims of the Study

Given these premises, we carried out a wide population-based cohort investigation aimed to assess the relationship between AP polytherapy (compared with AP monotherapy) and all-cause mortality in a large cohort of continuous AP users in Lombardy Region (Italy).

## 2. Materials and Methods

### 2.1. Setting

This study was based on the computerized healthcare utilization (HCU) databases of Lombardy, a region in northern Italy accounting for nearly 9.9 million inhabitants in 2018 (according to the Italian Institute of Statistics, https://demo.istat.it/, accessed on 21 March 2024), about 16% of the whole national population.

In Italy, all resident individuals have equal access to the healthcare services provided by the NHS and, in Lombardy as for each Italian region, a system of automated HCU databases has been implemented for the local management of the healthcare and its provision to residents. Therefore, HCU databases include data on all health services supplied (fully, or in part) free-of-charge by the NHS to residents, and collect a range of information, such as socio-demographics (gender, age, education level, etc.), inpatient or outpatient drug prescriptions (classified according to the Anatomical Therapeutic Chemical, ATC, classification system), and diagnosis at discharge from public or private hospitals (with diagnoses and procedures coded according to the International Classification of Diseases, 9th Revision, Clinical Modification, ICD-9-CM), all reimbursable by the NHS. Furthermore, a unique anonymous identification code for each NHS beneficiary is recorded and used in all databases. Then, by using this individual identification code, it is possible to perform a record-linkage procedure and interconnect these regional HCU databases, enabling one to study the complete healthcare path of each NHS beneficiary. Data are registered and stored according to the Italian and European General Data Protection Regulation, and more detailed information on the use of HCU databases in the field of pharmacoepidemiology and mental healthcare is available in previous publications [26,31,32,33]. Therapeutic (ATC) codes used for drawing records and fields from the considered databases are reported in Supplementary Table S1.

### 2.2. Target Population and Cohorts’ Selection

The target population included all NHS beneficiaries aged 18–79 years on 1st January 2018 living in the catchment areas of four local health units (LHUs) in Lombardy: Città Metropolitana of Milano, Sondrio, Brescia, Pavia. According to the Italian Institute of Statistics, the population covered by these 4 LHUs amounted to nearly 4.3 million people in 2018 and represent 56% of the entire adult population of Lombardy (https://demo.istat.it/, accessed on 21 March 2024).

According to the user-only paradigm [37], among these beneficiaries of RHS, those who, between 1 January and 31 December 2018, have received at least one prescription for antipsychotic drugs were identified. These patients represented the initial study cohort and were labelled as “Exposed to AP”. The date of the first antipsychotic drug prescription experienced in the inclusion period was defined as the “index date”.

For each patient included in the “exposed to AP” cohort, all the AP prescriptions dispensed during the follow-up were identified (for therapeutic codes, please see Supplementary Table S1). Because, in the HCU database, the information related to the prescribed daily doses (PDDs) is not recorded, the period covered by each prescription was calculated according to the defined daily dose (DDD) metric. DDDs were assigned and reviewed in accordance with the methodology developed by the WHO Collaborating Centre for Drug Statistics Methodology (https://www.whocc.no/atc_ddd_index/, accessed on 12 March 2022). For overlapping prescriptions, the patient was assumed to have taken all the drug(s) contained in the former prescription before starting the latter one.

**Figure 1:**
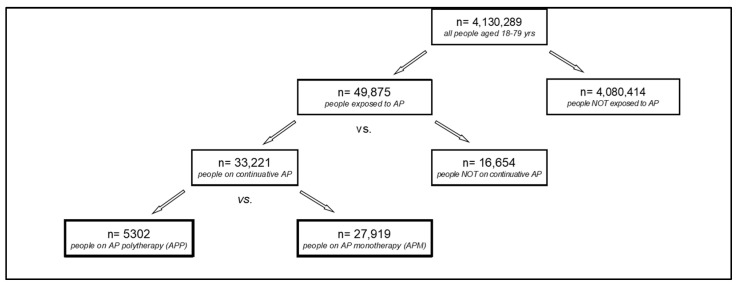
Flow-chart showing inclusion and exclusion criteria for the cohort selection process. Lombardy, Italy, 2018–2020.

According to Figure 1, with a description of the cohort selection process, the patients exposed to AP included in the initial study cohort were classified as “Continuous AP users” and “Non-continuous AP users”. Patients exposed to AP were considered as “continuous AP users” if they had been prescribed six or more antipsychotic different packages. Otherwise, if a patient received prescriptions for less than five antipsychotic different packages, he/she was considered as a “non-continuous AP user”.

Among the patients belonging to the “Continuous AP users” group, the distinction between the two groups of “Continuous AP monotherapy users” (APM) and “Continuous AP polytherapy users” (APP) was made. For continuous users, polytherapy (or APP) was defined according to the presence of two criteria: (a) the combined prescription of more than one AP [21]; and (b) a treatment overlap of at least two different APs for more than 30 days. The presence of both these two criteria was assumed as a likely proxy of a persistent and combined use of more than two different AP classes, i.e., antipsychotic polypharmacy.

### 2.3. Covariates

Baseline characteristics of cohort members included sex, age, and the presence of several comorbidities and/or co-treatments, as detected from drug prescriptions. Indeed, metabolic disturbances were detected from dispensations of antihypertensives, drugs used in diabetes, and lipid-modifying agents. The concomitant prescription of at least two classes of these medications was used as a proxy of metabolic syndrome (MS). Moreover, the prescription of at least six packages of antidiabetic medications was used as proxy indicator of the presence of diabetes mellitus (DM). Furthermore, each AP dispensation was converted in equivalent doses of olanzapine using the DDD method (on the basis of the international consensus study of antipsychotic dosing) [15,38] and, for each subject, the total AP dosage amount was calculated.

### 2.4. Outcome and Follow-Up

The occurrence of death for any cause was considered as the outcome of interest. Starting from the index date (i.e., start of follow-up), the patients included in the study accumulated person-time of observation, and overall survival (OS) was calculated until censoring (i.e., the earliest among death, migration, or 31 December 2020). Indeed, during the observation period, the included individuals left the study if they experienced the outcome of interest (in this case, the time when the event occurred was recorded) or a censorship (subjects for which the event of interest was not observed during the follow-up were defined as censored). Indeed, some individuals (i) did not develop the event during the study observation period (administrative censorship, endpoint fixed on 31 December 2020); some (ii) were lost to follow-up (non-informative censorship, e.g., change of region of residence); and others (iii) left the study for events other than the event of interest (informative censorship).

### 2.5. Statistical Analyses

The baseline characteristics of patients were described using summary statistics, with continuous variables being shown as mean ± standard deviation (SD) and categorical variables being described with relative percentages. Differences in the distribution of qualitative, and continuous, variables between exposure categories were analyzed using χ^2^ statistics and the *t*-test, respectively.

**Figure 2:**
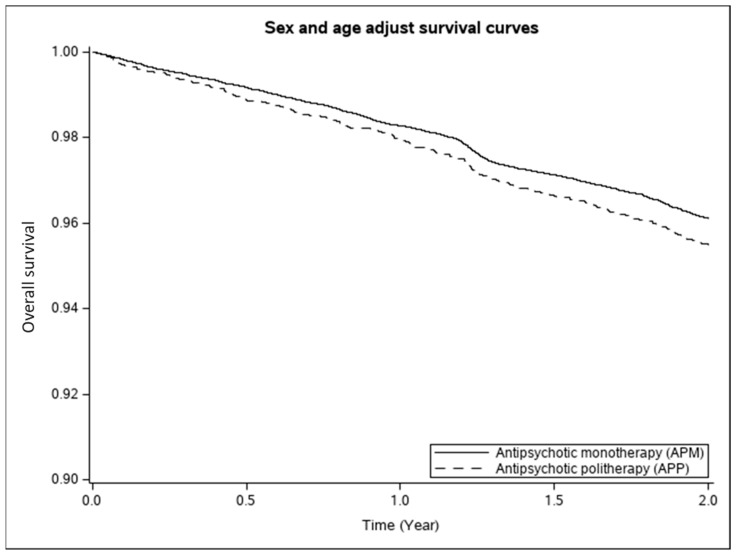
Comparison between Kaplan–Meier overall survival curves of continuous antipsychotic users on monotherapy (APM, n = 27,919) or polytherapy (APP, n = 5302). Lombardy, Italy, 2018–2020. Footnote: Overall survival (OS) estimated according to the Kaplan–Meier estimator. Adjustments were made for age and sex.

Survival curves comparing the groups of AP therapy were estimated using the method proposed by Kaplan–Meier. Therefore, the age- and sex-adjusted Kaplan–Meier overall survival (OS) curves of continuous AP users on monotherapy or polytherapy were estimated and compared (Figure 2). Moreover, the statistical comparison of the survival curves between the two groups (AP polytherapy and AP monotherapy) was performed by means of the log-rank test (Figure 2). In particular, the null hypothesis that there was no difference between the survival curves of the two groups, versus the alternative that there is at least one difference, was tested.

The association between AP polytherapy (compared with AP monotherapy, i.e., the reference category) and all-cause mortality was assessed using Cox regression models. As a preliminary step, the proportionality of hazards assumption for the time-fixed covariates was verified by means of Schoenfeld residuals [39,40], and the non-significant result (*p* > 0.050) suggested that the proportionality assumption had been met and holds approximately. Therefore, hazard ratios (HRs) and 95% corresponding confidence intervals (CIs) were calculated by univariate and multivariate Cox proportional hazard models. PH Cox regression models were adjusted for several potential confounders, identified from the existing literature, rather than using statistical criteria [41]. Confounders included in multivariate models were the above-listed covariates (please see the “Covariates” subsection): age; sex; presence of metabolic syndrome at baseline (yes or no); presence of diabetes mellitus at baseline (yes or no); total AP dosage amount. Although we adjusted for a wide range of confounders, residual confounding could not be completely ruled out.

*p*-values of less than 0.05 were considered to be statistically significant. Statistical analyses were performed using SAS software (version 9.4, SAS Institute, Cary, NC, USA).

## 3. Results

### 3.1. Patients’ Characteristics

As shown in the study flow-chart (Figure 1), a total of 49,875 patients aged 18–79 received at least one dispensation of antipsychotics during 2018 and were enrolled in the study (“Exposed to AP”). Of these, 33,221 were on a continuative treatment with antipsychotics and were classified as “Continuous AP users”. Among them, 5302 patients received APP treatment, whereas 27,919 constituted the APM group.

Baseline demographic and clinical characteristics of the included patients in accordance with AP therapy are reported in Table 1. Subjects on APM were mostly female and older; in particular, 41.5% of APM patients vs. 28.3% of those in the APP group were 60 years or older. The mean prescribed AP equivalent dose was nearly doubled for the APP group with respect to the APM one. Indeed, the values were 1.02 kg of olanzapine per-year (daily estimated dose of 2.78 mg) for the APM group and 2.36 kg of olanzapine (6.46 mg, daily) for APP. At baseline, APM patients had a higher use of lipid-lowering agents than APP ones, whereas no differences were found for antihypertensive drugs, antidiabetic agents, and the presence of metabolic disorders.

Concerning the group of patients with APP, a set of multiple combinations of different classes of AP were considered (Supplementary Table S2). Quetiapine was the most frequently prescribed medication, with almost five APP patients out of ten (44.6%) receiving at least one AP combination with this active principle. Furthermore, AP combinations with clozapine were also frequent, being prescribed to 589 (11.1%) patients with APP; among these combinations, the most frequent ones were clozapine with aripiprazole or haloperidol.

### 3.2. Kaplan–Meier Survival Analysis and Cox Regression Analysis of Survival

As also reported in Table 1, during the 2-year follow-up, 1958 (5.9%) subjects of the study cohort deceased. Overall, 1702 (6.1%) subjects died in the APM group and 256 (4.8%) in the APP group. Among these subjects experiencing death, those on APP were younger and prescribed with an AP equivalent dose double the APM ones (as reported in Supplementary Table S3, describing the baseline characteristics of all cases of death according to AP therapy).

Indeed, as showed in Figure 2 and reported in Table 2 with the results of the Cox PH regression model for this comparison, patients with APP showed an adjusted risk for all-cause mortality increased by 17% (95% CI: 2%; 33%) with respect to patients treated with APM.

Women and younger individuals were associated with lower risk for all-cause mortality, with hazard ratios of 0.49 (0.45–0.54) and 1.10 (1.09–1.11), respectively. Concerning other baseline covariates, an increment of 1 kg in the total AP dosage amount per year was associated with a 2% risk increase for all-cause mortality, whereas the presence of metabolic disorders exhibited an 19% increased risk, with an HR equal to 1.19 (1.04–1.35).

## 4. Discussion

Our study examines the overall survival in patients treated with AP polytherapy compared to patients treated with AP monotherapy. Results show that subjects prescribed with APP had a mortality risk that was nearly 20% significantly higher than those treated with APM. Among the considered covariates, the total AP dosage and the presence of metabolic disorders were associated with an increased risk for all-cause mortality, whereas lower risk was observed for female and younger individuals.

These findings suggest that the complexities of antipsychotic polypharmacy risk management transcend mere dosage and comorbidity considerations. They emphasize the necessity for an accurate assessment of polypharmacy, considering the potential synergistic effects of multiple drugs, which could play a critical role in increased mortality.

To better understand the results obtained, it is necessary to examine the study strengths and limitations. The present study is based on data from a large, unselected population, and can be reasonably defined as “population-based” offering guarantees of representativeness and generalizability. Indeed, using high-quality interconnectable individual data (as for HCU databases) on outpatient and inpatient services provided by the RHS, we were able to include all beneficiaries who were treated by public services, thereby generating reliable real-world evidence reflecting routine clinical practice, without reasonably being affected by recall biases or selective participation [42]. Therefore, our results can be generalized to the current situation of people treated with AP living in high-income countries with prevalent Caucasian populations and with free-of-charge national health service. Furthermore, we adopted some specific methodological precautions, such as the user-only paradigm [37], for controlling confounding. Nevertheless, as an observational study based on HCU data, confounding cannot be entirely ruled out, and future investigations are needed.

The reliance on HCU databases, while providing robust and large-scale real-world evidence, introduces specific limitations that merit careful consideration. First of all, the process of prescribing antipsychotic medications varies significantly across practitioners and is influenced by various factors, including the patient’s age, sex, physical health condition, and comorbidity. Indeed, it should be considered that our investigation lacks relevant data, such as diagnosis, clinical severity, socioeconomic status, and lifestyle habits (i.e., smoking status), which are all factors that could potentially influence both the selection of antipsychotic treatment strategy (APP versus APM) and the associated prognosis. However, the results were adjusted for several comorbidities and co-treatments, and it can be reasonably excluded that a worse outcome in the APP group might be due to the presence of such comorbidities or co-treatments. Despite these adjustments and other methodological instances of shrewdness helping to control for unmeasured confounding, further evidence investigating the influence of other socio-demographic and clinical variables is needed. Indeed, the absence of information on diagnosis constrains our ability to assess the appropriateness of antipsychotic prescriptions and to differentiate between patients receiving treatment for psychosis from those treated for conditions like resistant depression or dementia. Moreover, using HCU databases, drug use is based on the assumption that the purchase of a drug corresponds to its consumption. Despite this, it should also be considered that HCU data were originally established for recording all payments of healthcare providers to obtain reimbursement. Indeed, they are used to reimburse healthcare providers, where an incomplete or incorrect reporting leads to legal consequences, therefore assuring a high quality for the data source [26].

Lastly, as our study was observational, causality could not be determined, and we cannot rule out residual confounding. We did, however, adjust for a wide range of potential confounders. Another weakness is the limited external validity of our findings, as the cohort was not representative of the general population. The number of observed deaths was small, and we acknowledge that some analyses can be underpowered. Thus, to ensure that the results obtained can be generalized, further analyses need to be conducted on populations with different characteristics.

Despite the acknowledged limitations, our investigation adds valuable insights to the ongoing discourse on the safety and efficacy of antipsychotic treatment strategies, and our results are consistent with our initial expectations. Indeed, our analysis suggests that the higher mortality risk associated with antipsychotic polypharmacy is not fully accounted for by factors like antipsychotic dosage or metabolic disturbances alone. Our hypothesis, rooted in extensive evidence on the mental illness mortality gap and serious adverse effects associated with AP use, posited poorer outcomes with higher doses and polypharmacy [23,24,25,27,28,43,44,45,46,47]. Several observational studies have assessed the impact of these medications on general mortality rates for various diagnoses and age groups, and many of them found an association between AP use and mortality [48,49,50,51,52,53,54,55,56]. For instance, UK researchers analyzed nearly 11 million patients in primary care from 1995 to 2010, finding that antipsychotic users had almost tripled the mortality risk of non-users, with an adjusted standard mortality ratio (SMR) of 2.72 [57]. These results have been confirmed by several meta-analyses that showed evidence on the increased risk of death for some groups of AP users, particularly people with dementia [58,59].

Despite all these findings, the association between AP polytherapy (versus AP monotherapy) and all-cause mortality remains a contentious and unresolved issue in the literature [29]. Among the studies in the referenced inconclusive metanalysis, only one identified a lower mortality risk associated with APP [60]. However, since the study primary outcome was “all-cause discontinuation”, and it showed a lower outcome risk for APM, this incongruity raises concerns that selection bias might have significantly influenced mortality (secondary) outcomes in this research [60]. Another study by Kadra et al. (2018) examined the association between long-term APP and mortality using data from a large mental healthcare database. Their aim was to determine whether this risk varied based on the cause of death and antipsychotic dose [61]. Patients on long-term APP showed a slight, yet not consistently significant, elevated risk of mortality. This nuanced finding led the authors to suggest that while APP may entail a minor increased risk, the effect is not straightforward and varies depending on multiple factors, including the cause of death and dosage levels [61]. Bighelli et al. (2022) in their meta-analysis compared antipsychotic polypharmacy (APP) reduction to persistence in schizophrenia, focusing on outcomes like hospitalization, side effects, and, notably, mortality. The results showed no significant differences between the two approaches [62]. Furthermore, the study observed that persistence with APP might be associated with a lower rate of participant discontinuation due to inefficacy, emphasizing the critical need for personalized treatment plans and further investigation into the complex effects of APP. Conversely, our analysis highlights the critical need for a more cautious interpretation of APP benefits. While the results of Buhagiar et al. [29], Kadra et al. [61] and Bighelli et al. [62] contribute to the complex discourse on antipsychotic treatments, our findings emphasize the necessity of a more rigorous evaluation before endorsing APP as simply an equivalent treatment modality compared to APM, suggesting that its potential risks may outweigh the perceived benefits in many cases. This stance is not only informed by our analysis of mortality risks but also by a broader consideration of patient safety and treatment efficacy [48,49,50,51,52,53,54,55,56]. Furthermore, these findings are particularly crucial as a counterbalance to a number of recent observational studies that have emphasized the positive effect of second-generation antipsychotics, especially in long-acting formulations, on overall survival in schizophrenia [63,64,65,66,67,68]. This is important not only because critical views on their methodology have emerged, raising concerns [69,70], but also because it is crucial to consider that, overall, while treatment with AP may have a better impact on survival for patients with clearly severe and chronic disorders compared to no treatment at all, antipsychotics can have a negative effect, presumably due to side effects, on overall survival. This becomes even more evident with increasing doses or medication overlaps.

Finally, our study’s findings on the association between APP and increased mortality risk critically emphasize the need for a nuanced approach to antipsychotic therapy. The key clinical question should not merely pivot on whether APP is more effective or tolerable than APM, as the clinical practice inevitably gravitates toward employing APP in challenging cases. Instead, the focus should shift toward understanding the outcomes of deprescribing strategies, and particularly whether transitioning from APP to APM, following clinical stabilization, can improve physical health outcomes without compromising psychiatric stability, thereby potentially enhancing patient survival. Our results suggest the feasibility of designing pragmatic trials with moderate- to long-term follow-up, emphasizing that the observed differences between prescriptive modalities (APP versus APM) could become evident within manageable study sizes and within a relatively short observation period, as demonstrated by our findings at two years. Such studies could provide valuable insights into the relative risks for all-cause mortality associated with different antipsychotic treatment modalities, including deprescribing strategies. Thus, they could clarify deprescribing’s role in maintaining both physical health and psychiatric stability. Moreover, in light of these considerations, an immediate practical implication from our study suggests a cautious approach to long-term antipsychotic polypharmacy (APP), particularly for stabilized patients. Considering the associated increased mortality risk, clinicians might re-evaluate the necessity of APP versus the potential benefits of a gradual reduction or deprescribing. This suggestion aligns with personalized care and minimizes undue physical health risks.

## 5. Conclusions

Our analysis uncovered that continuous AP prescription and APP were significant predictors of vulnerability linked to a medium-term risk of all-cause mortality. This study highlighted the fact that the need for treatment strategies beyond pharmacology to address neuropsychiatric and physiological stressors, adopting a holistic approach, could ease antipsychotic treatment challenges in complex cases, potentially enhancing survival rates and life quality for individuals with psychotic spectrum disorders. Indeed, caution is recommended in routinely using antipsychotic polypharmacy in order to minimize the likelihood of adverse outcomes and death. Based on our findings, this study suggests the feasibility of designing pragmatic trials with moderate- to long-term follow-up periods. Such trials could offer valuable insights into the relative risks for all-cause mortality associated with different treatment modalities, including deprescribing.

## 6. Key Points

- Patients treated with antipsychotic polytherapy showed an adjusted mortality risk increased by 17% (95% CI: 2%; 33%) compared to antipsychotic monotherapy.
- This study highlights the potential risks associated with antipsychotic polypharmacy, emphasizing the importance of optimizing drug prescriptions to improve patient safety and reduce mortality rates in individuals receiving antipsychotic therapy.
- Further evidence investigating this topic, especially pragmatic trials with moderate- to long-term follow-up periods, is needed.

## Figures and Tables

**Table 1:** Main baseline demographic and clinico-pathological characteristics of cohort members in accordance with antipsychotic therapy. Lombardy, Italy, 2018–2020.

Factors		AP Continuous Users (N = 33,221)	AP Monotherapy, APM (N = 27,919)	AP Polytherapy, APP (N = 5302)	*p*-Value *
**Sex, *n* (%)**					
Females		16,575 (49.9)	14,210 (50.9)	2365 (40.6)	<0.0001
**Age, mean (SD)**		54.3 (15.6)	55.0 (15.7)	50.6 (14.7)	<0.0001
**Age classes (years), *n* (%)**					
	18–29	2635 (7.9)	2110 (7.6)	525 (9.9)	<0.0001
	30–39	3268 (9.8)	2663 (9.5)	605 (11.4)	
	40–49	6556 (19.7)	5224 (18.7)	1332 (25.1)	
	50–59	7679 (23.1)	6336 (22.7)	1343 (25.3)	
	60–69	6006 (18.1)	5122 (18.3)	884 (16.7)	
	70–79	7077 (21.4)	6464 (23.2)	613 (11.6)	
**Equivalent doses (kg), mean (SD)**		1.2 (3.0)	1.0 (2.7)	2.4 (4.3)	<0.0001
**LHU, *n* (%)**					
	Città Metropolitana di Milano	20,843 (62.7)	17,634 (63.3)	3209 (60.5)	<0.0001
	Sondrio	1730 (5.3)	1496 (5.4)	234 (4.4)	
	Brescia	7614 (22.9)	6135 (30.0)	1479 (27.9)	
	Pavia	3034 (9.1)	2654 (9.5)	380 (7.2)	
**Antidiabetics, *n* (%)**					
	1–5 packages	416 (1.3)	343 (1.3)	73 (1.4)	0.3783
	6+ packages	3253 (9.8)	2722 (9.7)	531 (10.0)	
**Antihypertensives ^¥^, *n* (%)**		637 (1.9)	536 (1.9)	101 (1.9)	0.9422
**Lipid modifying agents, *n* (%)**		6062 (18.2)	5171 (18.5)	891 (16.8)	0.0030
**Metabolic syndrome ^§^, *n* (%)**		2272 (6.8)	1926 (6.9)	346 (6.5)	0.3243
**All-cause deaths, *n* (%)**		1958 (5.9)	1702 (6.1)	256 (4.8)	0.0003

APs: antipsychotics; APM: antipsychotic monotherapy; APP: antipsychotic polytherapy; SD: standard deviation; LHUs: local health units. * *p*-value for the comparisons between APM vs. APP groups: χ^2^ test for categorical variables, or the Student’s *t*-test for the means of continuous variables. Statistically significant (at an alpha level of 0.05.) values in bold. ^¥^ Total AP dosage amount calculated converting each AP dispensation in equivalent doses of olanzapine using the DDD method (on the basis of the international consensus study of antipsychotic dosing). ^§^ The prescription of at least two previous classes of these medications was used as a proxy of the presence of metabolic syndrome.

**Table 2:** Multivariate Cox regression analysis of associations between type of therapy and type of use and all-cause mortality. Lombardy, Italy, 2018–2020.

		N	OS		
			HR ^¥^	95% CI	*p*-Value
**Type of AP therapy**		33,221			
	APM (ref.)		1.00	-	
	APP		1.17	1.02–1.33	**0.0223**
**Type of AP use**		49,875			
	AP non-continuative therapy (ref.)		1.00	-	
	AP continuative therapy		1.13	1.04–1.22	**0.0030**

OS: overall survival; HR: hazard ratio; CI: confidence interval. ^¥^ Hazard ratios (HRs) adjusted for age and sex. Significant *p*-values (*p* < 0.05) and HR values in univariate and multivariate survival analyses are bold.

## Data Availability

The data that support the findings of this study are available from the Region of Lombardy, but restrictions apply to the availability of these data, which were used under license for the current study, and so are not publicly available. More specifically, access to data was regulated by the EPIFARM agreement between the “Mario Negri” Institute for Pharmacological Research-IRCCS and the Regional Health Authority, and data were accessed obtaining authorization from the Regional Health Authority. Data are, however, available from the authors upon reasonable request and with permission of Lombardy Region, and further information on the results is available from the corresponding author on reasonable request.

## Supplementary Materials

The following supporting information can be downloaded at: https://www.mdpi.com/article/10.3390/jcm13072073/s1, Table S1: Therapeutic (ATC) codes used in the current study for drawing records and fields from healthcare utilization databases; Table S2: Antipsychotic combination of 5302 continuous AP polytherapy users (APP group) aged 18–79 years and living in the catchment areas of four LHU (Città Metropolitana di Milano, Sondrio, Brescia, Pavia) in 2018. Lombardy, Italy, 2018–2020; Table S3: Main baseline demographic and clinico-pathological characteristics of cohort members experiencing all-cause death in accordance with antipsychotic therapy. Lombardy, Italy, 2018–2020.

## References

[1] Verdoux H., Tournier M., Bégaud B. (2010). Antipsychotic Prescribing Trends: A Review of Pharmaco-Epidemiological Studies. Acta Psychiatr. Scand..

[2] Remington G., Addington D., Honer W., Ismail Z., Raedler T., Teehan M. (2017). Guidelines for the Pharmacotherapy of Schizophrenia in Adults. Can. J. Psychiatry Rev. Can. Psychiatr..

[3] Lehman A.F., Lieberman J.A., Dixon L.B., McGlashan T.H., Miller A.L., Perkins D.O., Kreyenbuhl J., American Psychiatric Association, Steering Committee on Practice Guidelines (2004). Practice Guideline for the Treatment of Patients with Schizophrenia, Second Edition. Am. J. Psychiatry.

[4] National Collaborating Centre for Mental Health (UK) (2009). Borderline Personality Disorder: Treatment and Management.

[5] Canadian Psychiatric Association Clinical Practice Guidelines (2005). Treatment of Schizophrenia. Can. J. Psychiatry Rev. Can. Psychiatr..

[6] National Collaborating Centre for Mental Health (UK) (2014). Psychosis and Schizophrenia in Adults: Treatment and Management: Updated Edition 2014.

[7] Scottish Intercollegiate Guidelines Network (SIGN) Management of Schizophrenia. https://www.sign.ac.uk/our-guidelines/management-of-schizophrenia/.

[8] Miller C., Bauer M.S. (2014). Excess Mortality in Bipolar Disorders. Curr. Psychiatry Rep..

[9] Ganguly R., Kotzan J.A., Miller L.S., Kennedy K., Martin B.C. (2004). Prevalence, Trends, and Factors Associated with Antipsychotic Polypharmacy among Medicaid-Eligible Schizophrenia Patients, 1998–2000. J. Clin. Psychiatry.

[10] Schumacher J.E., Makela E.H., Griffin H.R. (2003). Multiple Antipsychotic Medication Prescribing Patterns. Ann. Pharmacother..

[11] Stahl S.M. (1999). Antipsychotic Polypharmacy, Part 1: Therapeutic Option or Dirty Little Secret?. J. Clin. Psychiatry.

[12] Maher R.L., Hanlon J., Hajjar E.R. (2014). Clinical Consequences of Polypharmacy in Elderly. Expert Opin. Drug Saf..

[13] Loosbrock D.L., Zhao Z., Johnstone B.M., Morris L.S. (2003). Antipsychotic Medication Use Patterns and Associated Costs of Care for Individuals with Schizophrenia. J. Ment. Health Policy Econ..

[14] Stahl S.M. (2002). Antipsychotic Polypharmacy: Squandering Precious Resources?. J. Clin. Psychiatry.

[15] Leucht S., Samara M., Heres S., Davis J.M. (2016). Dose Equivalents for Antipsychotic Drugs: The DDD Method. Schizophr. Bull..

[16] Ceraso A., Lin J.J., Schneider-Thoma J., Siafis S., Tardy M., Komossa K., Heres S., Kissling W., Davis J.M., Leucht S. (2020). Maintenance Treatment with Antipsychotic Drugs for Schizophrenia. Cochrane Database Syst. Rev..

[17] Freudenreich O., Goff D.C. (2002). Antipsychotic Combination Therapy in Schizophrenia. A Review of Efficacy and Risks of Current Combinations. Acta Psychiatr. Scand..

[18] Yuzda M. (2000). Combination Antipsychotics: What Is the Evidence. J. Inf. Pharmacother..

[19] Lin S.-K. (2020). Antipsychotic Polypharmacy: A Dirty Little Secret or a Fashion?. Int. J. Neuropsychopharmacol..

[20] Lähteenvuo M., Tiihonen J. (2021). Antipsychotic Polypharmacy for the Management of Schizophrenia: Evidence and Recommendations. Drugs.

[21] Tiihonen J., Taipale H., Mehtälä J., Vattulainen P., Correll C.U., Tanskanen A. (2019). Association of Antipsychotic Polypharmacy vs. Monotherapy with Psychiatric Rehospitalization among Adults with Schizophrenia. JAMA Psychiatry.

[22] Srisurapanont M., Suttajit S., Maneeton N., Maneeton B. (2015). Efficacy and Safety of Aripiprazole Augmentation of Clozapine in Schizophrenia: A Systematic Review and Meta-Analysis of Randomized-Controlled Trials. J. Psychiatr. Res..

[23] Wastesson J.W., Morin L., Tan E.C.K., Johnell K. (2018). An Update on the Clinical Consequences of Polypharmacy in Older Adults: A Narrative Review. Expert Opin. Drug Saf..

[24] Scott I.A., Hilmer S.N., Reeve E., Potter K., Le Couteur D., Rigby D., Gnjidic D., Del Mar C.B., Roughead E.E., Page A. (2015). Reducing Inappropriate Polypharmacy: The Process of Deprescribing. JAMA Intern. Med..

[25] Salahudeen M.S. (2018). Deprescribing Medications in Older People: A Narrative Review. Drugs Today.

[26] Lora A., Monzio Compagnoni M., Allevi L., Barbato A., Carle F., D’avanzo B., Di Fiandra T., Ferrara L., Gaddini A., Leogrande M. (2022). The Quality of Mental Health Care Delivered to Patients with Schizophrenia and Related Disorders in the Italian Mental Health System. The QUADIM Project: A Multi-Regional Italian Investigation Based on Healthcare Utilisation Databases. Epidemiol. Psychiatr. Sci..

[27] Oakley P., Kisely S., Baxter A., Harris M., Desoe J., Dziouba A., Siskind D. (2018). Increased Mortality among People with Schizophrenia and Other Non-Affective Psychotic Disorders in the Community: A Systematic Review and Meta-Analysis. J. Psychiatr. Res..

[28] Stahl S.M., Mignon L., Meyer J.M. (2009). Which Comes First: Atypical Antipsychotic Treatment or Cardiometabolic Risk?. Acta Psychiatr. Scand..

[29] Buhagiar K., Templeton G., Blyth H., Dey M., Giacco D. (2020). Mortality Risk from Long-Term Treatment with Antipsychotic Polypharmacy vs Monotherapy among Adults with Serious Mental Illness: A Systematic Review and Meta-Analysis of Observational Studies. Schizophr. Res..

[30] Istat Ricostruzione Della Popolazione 2002–2019. https://demo.istat.it/app/?i=RIC&l=it.

[31] Monzio Compagnoni M., Caggiu G., Allevi L., Barbato A., Carle F., D’Avanzo B., Di Fiandra T., Ferrara L., Gaddini A., Giordani C. (2023). Assessment and Monitoring of the Quality of Clinical Pathways in Patients with Depressive Disorders: Results from a Multiregional Italian Investigation on Mental Health Care Quality (the QUADIM Project). J. Clin. Med..

[32] D’Avanzo B., Barbato A., Monzio Compagnoni M., Caggiu G., Allevi L., Carle F., Di Fiandra T., Ferrara L., Gaddini A., Sanza M. (2023). The Quality of Mental Health Care for People with Bipolar Disorders in the Italian Mental Health System: The QUADIM Project. BMC Psychiatry.

[33] Sanza M., Monzio Compagnoni M., Caggiu G., Allevi L., Barbato A., Campa J., Carle F., D’avanzo B., Di Fiandra T., Ferrara L. (2023). Assessing the Quality of the Care Offer for People with Personality Disorders in Italy: The QUADIM Project. A Multicentre Research Based on the Database of Use of Mental Health Services. Int. J. Ment. Health Syst..

[34] D’Mello C., Swain M.G. (2017). Immune-to-Brain Communication Pathways in Inflammation-Associated Sickness and Depression. Curr. Top. Behav. Neurosci..

[35] Hammond J.C., Shan D., Meador-Woodruff J.H., McCullumsmith R.E., Popoli M., Diamond D., Sanacora G. (2014). Evidence of Glutamatergic Dysfunction in the Pathophysiology of Schizophrenia. Synaptic Stress and Pathogenesis of Neuropsychiatric Disorders.

[36] Grillo C.A., Reagan L.P., Popoli M., Diamond D., Sanacora G. (2014). Metabolic Stress and Neuropsychiatric Disorders. Synaptic Stress and Pathogenesis of Neuropsychiatric Disorders.

[37] Corrao G., Ghirardi A., Segafredo G., Zambon A., Della Vedova G., Lapi F., Cipriani F., Caputi A., Vaccheri A., Gregori D. (2014). User-Only Design to Assess Drug Effectiveness in Clinical Practice: Application to Bisphosphonates and Secondary Prevention of Fractures. Pharmacoepidemiol. Drug Saf..

[38] Gardner D.M., Murphy A.L., O’Donnell H., Centorrino F., Baldessarini R.J. (2010). International Consensus Study of Antipsychotic Dosing. Am. J. Psychiatry.

[39] Sjölander A., Dickman P. (2024). Why Test for Proportional Hazards—Or Any Other Model Assumptions?. Am. J. Epidemiol..

[40] Grambsch P.M., Therneau T.M. (1994). Proportional Hazards Tests and Diagnostics Based on Weighted Residuals. Biometrika.

[41] Greenland S., Daniel R., Pearce N. (2016). Outcome Modelling Strategies in Epidemiology: Traditional Methods and Basic Alternatives. Int. J. Epidemiol..

[42] Corrao G., Monzio Compagnoni M., Barbato A., D’Avanzo B., Di Fiandra T., Ferrara L., Gaddini A., Saponaro A., Scondotto S., Tozzi V.D. (2022). From Contact Coverage to Effective Coverage of Community Care for Patients with Severe Mental Disorders: A Real-World Investigation from Italy. Front. Psychiatry.

[43] Harris E.C., Barraclough B. (1997). Suicide as an Outcome for Mental Disorders. A Meta-Analysis. Br. J. Psychiatry J. Ment. Sci..

[44] Saha S., Chant D., McGrath J. (2007). A Systematic Review of Mortality in Schizophrenia: Is the Differential Mortality Gap Worsening over Time?. Arch. Gen. Psychiatry.

[45] Lawrence D., Kisely S., Pais J. (2010). The Epidemiology of Excess Mortality in People with Mental Illness. Can. J. Psychiatry Rev. Can. Psychiatr..

[46] Laursen T.M., Munk-Olsen T., Vestergaard M. (2012). Life Expectancy and Cardiovascular Mortality in Persons with Schizophrenia. Curr. Opin. Psychiatry.

[47] Hjorthøj C., Stürup A.E., McGrath J.J., Nordentoft M. (2017). Years of Potential Life Lost and Life Expectancy in Schizophrenia: A Systematic Review and Meta-Analysis. Lancet Psychiatry.

[48] Basciotta M., Zhou W., Ngo L., Donnino M., Marcantonio E.R., Herzig S.J. (2020). Antipsychotics and the Risk of Mortality or Cardiopulmonary Arrest in Hospitalized Adults. J. Am. Geriatr. Soc..

[49] Calsolaro V., Antognoli R., Okoye C., Monzani F. (2019). The Use of Antipsychotic Drugs for Treating Behavioral Symptoms in Alzheimer’s Disease. Front. Pharmacol..

[50] Harrison S.L., Sluggett J.K., Lang C., Whitehead C., Crotty M., Corlis M., Wesselingh S., Inacio M.C. (2021). Initiation of Antipsychotics after Moving to Residential Aged Care Facilities and Mortality: A National Cohort Study. Aging Clin. Exp. Res..

[51] Hoang U., Stewart R., Goldacre M.J. (2011). Mortality after Hospital Discharge for People with Schizophrenia or Bipolar Disorder: Retrospective Study of Linked English Hospital Episode Statistics, 1999–2006. BMJ.

[52] Jayatilleke N., Hayes R.D., Chang C.-K., Stewart R. (2018). Acute General Hospital Admissions in People with Serious Mental Illness. Psychol. Med..

[53] Jennum P., Baandrup L., Ibsen R., Kjellberg J. (2015). Increased All-Cause Mortality with Use of Psychotropic Medication in Dementia Patients and Controls: A Population-Based Register Study. Eur. Neuropsychopharmacol..

[54] Ray W.A., Stein C.M., Murray K.T., Fuchs D.C., Patrick S.W., Daugherty J., Hall K., Cooper W.O. (2019). Association of Antipsychotic Treatment with Risk of Unexpected Death among Children and Youths. JAMA Psychiatry.

[55] Weintraub D., Chiang C., Kim H.M., Wilkinson J., Marras C., Stanislawski B., Mamikonyan E., Kales H.C. (2016). Association of Antipsychotic Use with Mortality Risk in Patients with Parkinson Disease. JAMA Neurol..

[56] Gerhard T., Stroup T.S., Correll C.U., Setoguchi S., Strom B.L., Huang C., Tan Z., Crystal S., Olfson M. (2020). Mortality Risk of Antipsychotic Augmentation for Adult Depression. PLoS ONE.

[57] Murray-Thomas T., Jones M.E., Patel D., Brunner E., Shatapathy C.C., Motsko S., Van Staa T.P. (2013). Risk of Mortality (Including Sudden Cardiac Death) and Major Cardiovascular Events in Atypical and Typical Antipsychotic Users: A Study with the General Practice Research Database. Cardiovasc. Psychiatry Neurol..

[58] Ralph S.J., Espinet A.J. (2018). Increased All-Cause Mortality by Antipsychotic Drugs: Updated Review and Meta-Analysis in Dementia and General Mental Health Care. J. Alzheimers Dis. Rep..

[59] Schneider L.S., Dagerman K.S., Insel P. (2005). Risk of Death with Atypical Antipsychotic Drug Treatment for Dementia: Meta-Analysis of Randomized Placebo-Controlled Trials. JAMA.

[60] Katona L., Czobor P., Bitter I. (2014). Real-World Effectiveness of Antipsychotic Monotherapy vs. Polypharmacy in Schizophrenia: To Switch or to Combine? A Nationwide Study in Hungary. Schizophr. Res..

[61] Kadra G., Stewart R., Shetty H., MacCabe J.H., Chang C.-K., Taylor D., Hayes R.D. (2018). Long-Term Antipsychotic Polypharmacy Prescribing in Secondary Mental Health Care and the Risk of Mortality. Acta Psychiatr. Scand..

[62] Bighelli I., Rodolico A., Siafis S., Samara M.T., Hansen W.-P., Salomone S., Aguglia E., Cutrufelli P., Bauer I., Baeckers L. (2022). Antipsychotic Polypharmacy Reduction versus Polypharmacy Continuation for People with Schizophrenia. Cochrane Database Syst. Rev..

[63] Correll C.U., Solmi M., Croatto G., Schneider L.K., Rohani-Montez S.C., Fairley L., Smith N., Bitter I., Gorwood P., Taipale H. (2022). Mortality in People with Schizophrenia: A Systematic Review and Meta-Analysis of Relative Risk and Aggravating or Attenuating Factors. World Psychiatry Off. J. World Psychiatr. Assoc. WPA.

[64] Tiihonen J., Lönnqvist J., Wahlbeck K., Klaukka T., Niskanen L., Tanskanen A., Haukka J. (2009). 11-Year Follow-up of Mortality in Patients with Schizophrenia: A Population-Based Cohort Study (FIN11 Study). Lancet.

[65] Cullen B.A., McGinty E.E., Zhang Y., Dosreis S.C., Steinwachs D.M., Guallar E., Daumit G.L. (2013). Guideline-Concordant Antipsychotic Use and Mortality in Schizophrenia. Schizophr. Bull..

[66] Torniainen M., Mittendorfer-Rutz E., Tanskanen A., Björkenstam C., Suvisaari J., Alexanderson K., Tiihonen J. (2015). Antipsychotic Treatment and Mortality in Schizophrenia. Schizophr. Bull..

[67] Taipale H., Mittendorfer-Rutz E., Alexanderson K., Majak M., Mehtälä J., Hoti F., Jedenius E., Enkusson D., Leval A., Sermon J. (2018). Antipsychotics and Mortality in a Nationwide Cohort of 29,823 Patients with Schizophrenia. Schizophr. Res..

[68] Taipale H., Tanskanen A., Mehtälä J., Vattulainen P., Correll C.U., Tiihonen J. (2020). 20-Year Follow-up Study of Physical Morbidity and Mortality in Relationship to Antipsychotic Treatment in a Nationwide Cohort of 62,250 Patients with Schizophrenia (FIN20). World Psychiatry Off. J. World Psychiatr. Assoc. WPA.

[69] Taylor R.W., Marwood L., Oprea E., DeAngel V., Mather S., Valentini B., Zahn R., Young A.H., Cleare A.J. (2020). Pharmacological Augmentation in Unipolar Depression: A Guide to the Guidelines. Int. J. Neuropsychopharmacol..

[70] Whitaker R. (2020). Viewpoint: Do Antipsychotics Protect against Early Death? A Critical View. Psychol. Med..

