# Supplementary material for: Association of Antipsychotic Polypharmacy and Two-Year All-Cause Mortality: A Population-Based Cohort Study of 33,221 Italian Continuous Users
Source: J Clin Med. 2024 Apr 3;13(7):2073. doi: 10.3390/jcm13072073 (PMC11012528; doi:10.3390/jcm13072073)
Supplement: Supplementary file 1 [file jcm-13-02073-s001.zip › jcm-2914483-supplementary.pdf]

Supplementary Table S1. Therapeutic (ATC) codes used in the current study for drawing records and fields from Healthcare Utilization databases.

	Coding system	Codes
**Drugs**		
Antipsychotics		
Phenothiazines with aliphatic sidechain	*ATC*	N05AA
Phenothiazines with piperazine structure	*ATC*	N05AB
Phenothiazines with piperidine structure	*ATC*	N05AC
Butyrophenone derivatives	*ATC*	N05AD
Ziprasidone	*ATC*	N05AE04
Thioxanthene derivatives	*ATC*	N05AF
Diphenylbutylpiperidine derivatives	*ATC*	N05AG
Clozapine	*ATC*	N05AH02
Olanzapine	*ATC*	N05AH03
Quietapine	*ATC*	N05AH04
Asenapine	*ATC*	N05AH05
Benzamides	*ATC*	N05AL
Risperidone	*ATC*	N05AX08
Aripiprazole	*ATC*	N05AX12
Paliperidone	*ATC*	N05AX13
Antihypertensives	*ATC*	C02
Antidiabetics	*ATC*	A10
Lipid modifying agents	*ATC*	C10

Supplementary Table S2. Antipsychotic combination of 5302 continuous AP polytherapy users (APP group), aged 18-79 years and living in the catchment areas of four LHU (*Città Metropolitana di Milano, Sondrio, Brescia, Pavia*) in 2018. Lombardy, Italy, 2018-2020.

	Quetiapine	Olanzapine	Aripiprazole	Haloperidol	Risperidone	Paliperidone
Quetiapine	-	422 (8.0%)	467 (8.8%)	357 (6.7%)	289 (5.5%)	122 (2.3%)
Olanzapine	422 (8.0%)	-	248 (4.7%)	236 (4.5%)	133 (2.5%)	79 (1.5%)
Aripiprazole	467 (8.8%)	248 (4.7%)	-	123 (2.3%)	114 (2.2%)	72 (1.4%)
Other APs	1476 (27.8%)	838 (15.8%)	714 (13.5%)	423 (8.0%)	358 (6.8%)	202 (3.8%)
Total	2365 (44.6%)	1508 (28.4%)	1429 (27.0%)	1139 (21.5%)	894 (16.9%)	475 (9.0%)

AP: Antipsychotics. The sum of percentages exceeds 100% due to some prescriptions being counted in more than one AP category.

Supplementary Table S3. Main baseline demographic and clinico-pathological characteristics of cohort members experiencing all-cause death, according with Antipsychotic therapy. Lombardy, Italy, 2018-2020.

	**All AP continuous users**	**AP Monotherapy, APM**	**AP Polytherapy, APP**	*p-value**
**Deaths for any-cause, N**	1958	1702	256	
**Sex, *n* (%)**				
Females	813 (41.5)	707 (41.5)	106 (41.4)	0.2426
**Age, mean (SD)**	70.8 (9.9)	71.3 (9.3)	69.1 (11.5)	0.0427
**Age classes (years), *n* (%)**				**<0.0001**
18-29	14 (0.7)	10 (0.6)	4 (1.6)	
30-39	26 (1.3)	20 (1.2)	6 (2.3)	
40-49	102 (5.2)	77 (4.5)	25 (9.8)	
50-59	221 (11.3)	179 (10.5)	42 (16.4)	
60-69	373 (19.1)	305 (17.9)	68 (26.6)	
70-79	1222 (62.4)	1111 (65.3)	111 (43.4)	
**Equivalent doses (kg), *mean* (SD)**	0.7 (1.0)	0.7 (0.8)	1.2 (1.6)	**<0.0001**
**LHU, *n* (%)**				0.5859
Città Metropolitana di Milano	1206 (61.6)	1054 (61.9)	152 (59.4)	
Sondrio	140 (7.2)	116 (6.8)	24 (9.4)	
Brescia	335 (17.1)	290 (17.0)	45 (17.6)	
Pavia	278 (14.2)	242 (14.2)	36 (14.1)	
**Antidiabetics, *n* (%)**				0.5859
1-5 packages	59 (3.0)	47 (2.8)	12 (4.7)	
6+ packages	394 (20.1)	337 (19.8)	57 (22.3)	
**Antihypertensives ¥, *n* (%)**	62 (3.2)	47 (2.8)	15 (5.9)	0.1700
**Lipid modifying agents, *n* (%)**	457 (23.3)	400 (23.5)	57 (22.3)	0.7792
**Metabolic syndrome §, *n* (%)**	195 (10.0)	174 (10.2)	21 (8.2)	0.5644
**Mean time of follow-up, *mean* (SD)**	561.7 (182.3)	565.4 (182.1)	547.9 (183.7)	0.4289

* P-value for the comparisons between APM vs APP groups: $\chi 2$ test for categorical variables, or the Student's t-test for the means of continuous variables ; ** Statistically significant (at an *alpha* level of 0.05.) values in bold.

£ The prescription of at least two previous classes of these medications in was used as a proxy of the presence of metabolic syndrome.

¥ Total AP dosage amount calculated converting each AP dispensation in equivalent doses of olanzapine using the DDD method (on the basis of the international consensus study of antipsychotic dosing; LHU Local Health Units.
